# Integration of neutrophil to high-density lipoprotein ratio for prognostic assessment of nasopharyngeal carcinoma: a large-scale long-term retrospective study

**DOI:** 10.3389/fphys.2026.1729581

**Published:** 2026-04-07

**Authors:** Nan-Jun Chen, Xu-Xin Lin, Rui-Xin Cheng, Ao-Qiang Chen, Yong-Miao Lin, Zhi-Qing Long, Fang-Fang Duan, Xin Huang, Wen Xia, De-Huan Xie, Yuan Lei, Sha-Sha Du, Chen Ren, Xin Hua

**Affiliations:** 1 Department of Radiation Oncology, Guangdong Provincial People’s Hospital (Guangdong Academy of Medical Sciences), Southern Medical University, Guangzhou, China; 2 School of Medicine South China University of Technology, Guangzhou, China; 3 Sun Yat-sen University Cancer Center, State Key Laboratory of Oncology in South China, Guangdong Key Laboratory of Nasopharyngeal Carcinoma Diagnosis and Therapy, Guangdong Provincial Clinical Research Center for Cancer, Guangzhou, China

**Keywords:** concurrent chemoradiotherapy (CCRT), nasopharyngeal carcinoma, NHR, overall survival (OS), prognosis

## Abstract

**Background:**

Recent research suggests that the emerging neutrophil to high-density lipoprotein ratio (NHR) has a significant correlation with the survival outcomes across a range of tumors. However, its prognostic value in nasopharyngeal carcinoma (NPC) remained to be elucidated. This study aimed to evaluate the relationship between NHR and overall survival (OS) in patients with NPC, as well as to develop a corresponding prognostic model.

**Methods:**

We conducted a retrospective analysis of 834 NPC patients who received platinum-based concurrent chemoradiotherapy (CCRT) between January 2010 and December 2014. The optimal NHR cutoff was determined using maximally selected rank statistics. Survival analyses were performed using Kaplan-Meier methods and Cox regression models. A prognostic nomogram incorporating NHR with conventional risk factors was developed and validated.

**Results:**

The NHR score successfully segregated NPC patients into two categories with significantly different OS (HR = 0.672, 95% CI: 0.481–0.939, P = 0.019). Multivariate analysis confirmed NHR as an independent prognostic factor, along with age, T stage, and N stage. Considering EBV-DNA is a well-established prognostic factor for NPC in accordance with clinical practice and previous studies, we incorporated it together with the above factors to construct the prognostic nomogram. The established nomogram showed modestly higher predictive accuracy (C-index = 0.696, 95% CI: 0.654–0.738) compared to conventional staging parameters (C-index = 0.648, 95% CI: 0.583–0.713).

**Conclusion:**

Pre-treatment NHR serves as an independent prognostic indicator for survival in NPC patients undergoing CCRT. This readily available, cost-effective biomarker, when integrated with traditional prognostic factors, provides a comprehensive tool for risk stratification and treatment planning. External validation through multicenter studies is warranted to confirm these findings.

## Introduction

More than 130,000 new nasopharyngeal carcinoma (NPC) cases are reported worldwide annually, with over 70% presenting as locoregionally advanced disease (Liu et al., 2025). Given the tumor’s notable radiosensitivity, concurrent chemoradiotherapy (CCRT) has emerged as the cornerstone treatment for locally advanced NPC, demonstrating superior outcomes as treatment modalities have evolved from two-dimensional radiotherapy to intensity-modulated radiotherapy (Miao et al., 2022; Zhang et al., 2019; Juarez-Vignon Whaley et al., 2023). While the Union for International Cancer Control/American Joint Committee on Cancer (UICC/AJCC) TNM staging system remains the gold standard for predicting prognosis and guiding treatment decisions, significant heterogeneity in clinical outcomes exists among patients with identical TNM stages, with up to 30% experiencing disease progression despite similar treatment regimens (Rai et al., 2025; Chiang et al., 2021; Liu et al., 2024). This discrepancy stems from the TNM system’s exclusive focus on anatomical tumor invasion, without consideration of tumor cell functional status or systemic factors, highlighting the urgent need for complementary prognostic markers (Liu et al., 2022; Tang et al., 2022; Pan et al., 2016a; Blanchard et al., 2025).

In recent years, the complex interplay between inflammation and cancer progression has gained increasing attention, with chronic inflammatory processes recognized as key modulators of the tumor microenvironment and drivers of carcinogenesis (Mantovani et al., 2008; Grivennikov et al., 2010; Hanahan and Weinberg, 2011). This understanding has led to the investigation of various systemic inflammation markers, including neutrophil-to-lymphocyte ratio (NLR), platelet-to-lymphocyte ratio (PLR), and systemic immune-inflammation index (SII), which have shown significant prognostic value in multiple cancer types including NPC (Yuan et al., 2023; Wang et al., 2022; Huang et al., 2020; Templeton et al., 2014). Parallel research has revealed the critical role of dysregulated cholesterol metabolism in tumor pathogenesis, particularly through high-density lipoprotein (HDL), which exhibits anti-inflammatory, antioxidant, and immunomodulatory properties beyond its traditional role in cholesterol transport (Kim et al., 2024; Zhou et al., 2018; Mai et al., 2023). The integration of these two pathways through the neutrophil to high-density lipoprotein ratio (NHR) offers a novel approach to patient assessment, combining both inflammatory and metabolic parameters in a single, easily obtainable marker (Shi et al., 2023; Peng et al., 2024; Wang et al., 2021).

Given the limitations of current prognostic tools and the promising potential of integrated biomarkers, this study aims to investigate the prognostic significance of NHR in NPC patients receiving CCRT. By examining the relationship between NHR and patient outcomes, we seek to address the critical gap in current staging systems that rely solely on anatomical parameters. Furthermore, we aim to develop a novel predictive model incorporating NHR for individualized survival predictions, potentially offering a more comprehensive approach to risk stratification. This research could enhance clinical decision-making by providing additional tools for patient assessment and treatment planning, ultimately contributing to more personalized therapeutic strategies for NPC patients.

## Methods

### Patients

This retrospective study enrolled consecutive NPC patients who received platinum-based CCRT between January 2010 and December 2014 at Sun Yat-sen University Cancer Center located in endemic regions of China. The inclusion criteria were: (i) histologically confirmed non-metastatic NPC without prior treatment; (ii) complete pre-treatment peripheral blood tests including neutrophil count and HDL measurements; (iii) treatment with radical intensity-modulated radiotherapy combined with concurrent platinum-based chemotherapy; and (iv) absence of chronic inflammatory conditions or lipid-lowering medication use. All patients were staged according to the 8th edition of the AJCC TNM staging system. The study protocol was approved by the Sun Yat-sen University Cancer Center Ethics Committee, which waived the requirement for written informed consent due to the retrospective nature of the study. All procedures were conducted in accordance with the ethical standards of the 1964 Declaration of Helsinki and its subsequent amendments.

### Data collection and follow-up

Baseline laboratory data were collected within 1 week before treatment initiation. The NHR was calculated by dividing the absolute neutrophil count by the HDL level. Clinical and pathological data were extracted from electronic medical records. Body mass index (BMI) was calculated as weight (kg)/height (m)^2^, with patients categorized as obese (BMI ≥28 kg/m^2^), overweight (24 kg/m^2^ < BMI <28 kg/m^2^), or normal weight (BMI ≤24 kg/m^2^). Treatment protocols followed standard institutional guidelines for NPC management. Overall survival (OS) was defined as the time from diagnosis to death from any cause or last follow-up.

### Statistical analysis

The optimal cutoff value for NHR was determined using maximally selected rank statistics with OS as the endpoint through the “maxstat” package, we have incorporated bootstrapping validation (n = 1,000) to evaluate the stability of the cutoff value of NHR. Survival curves were generated using the Kaplan-Meier method and compared using log-rank tests. The proportional hazards assumption was verified using Schoenfeld residuals. Univariate Cox regression identified potential prognostic factors (P < 0.05), which were entered into multivariate Cox models to determine independent predictors. A prognostic nomogram was constructed based on independent risk factors identified in the training cohort. The model’s performance was evaluated using Harrell’s concordance index (C-index), time-dependent Receiver Operating Characteristic (tROC), and decision curve analysis (DCA). The nomogram’s predictive accuracy was assessed through calibration curves. Statistical analyses were performed using R software version 4.2.1, with two-sided P < 0.05 considered statistically significant.

## Results

### Patient characteristics

This study included 834 patients with nasopharyngeal carcinoma. The cohort comprised 617 (74.00%) male and 217 (26.00%) female patients. The age distribution showed 411 (49.30%) patients >45 years and 423 (50.70%) patients ≤45 years. Most patients (98.40%) had WHO type III histology. Regarding T stage distribution, 42 (5.04%) patients were T1, 160 (19.20%) T2, 507 (60.80%) T3, and 125 (15.00%) T4. For N stage, 77 (9.23%) were N0, 448 (53.70%) N1, 265 (31.80%) N2, and 44 (5.28%) N3. EBV-DNA levels were ≥4000 copies/mL in 269 (32.30%) patients. The optimal cutoff value of NHR was identified as 2.557. Bootstrapping validation with 1000 resamples for this threshold yielded a corrected C-statistic of 0.816, confirming its favorable stability and internal validity. NHR≤2.557 was observed in 78.50% (Table 1).

**TABLE 1:** Patient demographics and clinical characteristics.

Characteristic	No. (%) of Patients
Age	
>45 years	411 (49.3%)
≤45 years	423 (50.7%)
Gender	
Female	217 (26.0%)
Male	617 (74.0%)
Histological type	
WHO I/II	13 (1.56%)
WHO III	821 (98.4%)
HGB	
<113 g/L	26 (3.12%)
113–151 g/L	535 (64.1%)
≥151 g/L	273 (32.7%)
LDH	
≥245 U/L	50 (6.00%)
<245 U/L	784 (94.0%)
T stage	
T1	42 (5.04%)
T2	160 (19.2%)
T3	507 (60.8%)
T4	125 (15.0%)
N stage	
N0	77 (9.23%)
N1	448 (53.7%)
N2	265 (31.8%)
N3	44 (5.28%)
BMI	
≤24 kg/m^2^	505 (60.6%)
24–28 kg/m^2^	282 (33.8%)
≥28 kg/m^2^	47 (5.64%)
EBV-DNA	
<4000 copies/mL	565 (67.7%)
≥4000 copies/mL	269 (32.3%)
ALB	
≥40 g/L	765 (91.8%)
<40 g/L	68 (8.16%)
NHR	
≤2.557	655 (78.5%)
>2.557	179 (21.5%)

Abbreviations: WHO, World Health Organization; HGB, hemoglobin; LDH, serum lactate dehydrogenase levels; EBV-DNA, Epstein-Barr virus DNA; NHR, neutrophil to high-density lipoprotein ratio.

### Prognostic value of NHR score for OS in NPC

**FIGURE 1:**
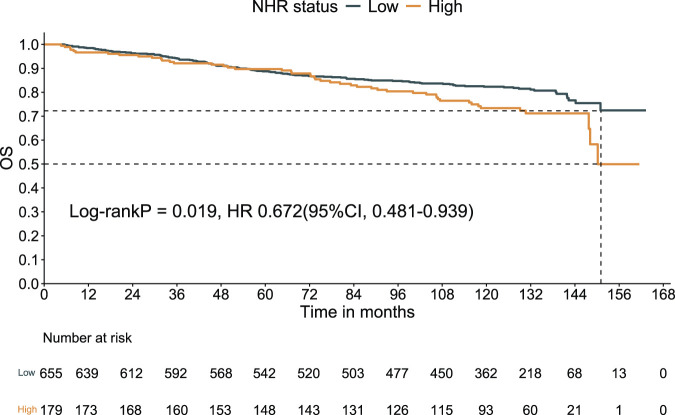
Kaplan-Meier survival curves for patients stratified by NHR groups. Abbreviations: NHR, neutrophil to high-density lipoprotein ratio; HR, hazard ratios; CI, confidence interval.

Following the determination and validation of the optimal NHR cutoff value, the prognostic significance of pre-treatment NHR for OS in the enrolled NPC cohort was further evaluated through survival analysis. Median OS was 123.2 months (IQR:88.1–136.1), with 168 deaths recorded. The 1-, 3-, and 5-year OS rates were 98.1%, 93.8%, and 89.0% respectively. Patients in the low-NHR group (NHR ≤2.557) had better survival outcomes than those in the high-NHR group (NHR>2.557) (HR = 0.672; 95% CI: 0.481–0.939; P = 0.019; Figure 1).

### Univariate and multivariate cox regression analyses of OS in NPC

To identify independent prognostic factors for OS in NPC patients treated with concurrent chemoradiotherapy, univariate and subsequent multivariate Cox proportional hazards regression analyses were conducted with clinically relevant variables. In univariate analysis, several factors were significantly associated with overall survival: NHR, age, T stage, N stage, EBV-DNA (see Table 2). After adjustment, NHR, age, advanced T stage (T3/T4) and nodal involvement (N1–N3) remained independent predictors of poorer OS.

**TABLE 2:** Univariate and multivariate Cox regression analyses of overall survival.

Characteristic	Univariate analysis		Multivariate analysis	
	Hazard ratio (95% CI)	P	Hazard ratio (95% CI)	P
Age				
>45 years	1		1	
≤45 years	0.562 (0.411–0.767)	<0.001	0.553 (0.405–0.756)	<0.001
Gender				
Female	1			
Male	1.102 (0.777–1.564)	0.585		
Histological type				
WHO I/II	1			
WHO III	0.447 (0.184–1.089)	0.077		
HGB				
<113 g/L	1			
113–151 g/L	1.874 (0.595–5.903)	0.283		
≥151 g/L	1.813 (0.567–5.801)	0.316		
LDH				
≥245 U/L	1			
<245 U/L	0.671 (0.381–1.181)	0.167		
T stage				
T1	1		1	
T2	3.749 (0.890–15.800)	0.072	3.307 (0.784–13.958)	0.104
T3	4.682 (1.155–18.990)	0.031	4.422 (1.088–17.978)	0.038
T4	9.007 (2.178–37.250)	0.002	8.842 (2.128–36.749)	0.003
N stage				
N0	1		1	
N1	2.523 (1.102–5.775)	0.029	2.814 (1.223–6.475)	0.015
N2	3.400 (1.472–7.852)	0.004	4.005 (1.717–9.345)	0.001
N3	4.695 (1.782–12.367)	0.002	5.259 (1.961–14.105)	<0.001
BMI				
≤24 kg/m^2^	1			
24–28 kg/m^2^	0.718 (0.511–1.009)	0.057		
≥28 kg/m^2^	0.842 (0.427–1.661)	0.620		
EBV-DNA				
<4000 copies/mL	1		1	
≥4000 copies/mL	1.531 (1.124–2.086)	0.007	1.246 (0.903–1.720)	0.180
ALB				
≥40 g/L	1			
<40 g/L	1.562 (0.957–2.549)	0.074		
NHR				
≤2.557	1		1	
>2.557	1.488 (1.065–2.079)	0.020	1.560 (1.114–2.185)	0.010

Hazard ratios estimated by Cox proportional hazards regression. All statistical tests were two-sided.

Abbreviations: WHO, world health organization; HGB, hemoglobin; LDH, serum lactate dehydrogenase levels; BMI, body mass index; EBV-DNA, Epstein-Barr virus DNA; ALB, albumin; NHR, neutrophil to high-density lipoprotein ratio.

### Development of the integrated prognostic model based on NHR

**FIGURE 2:**
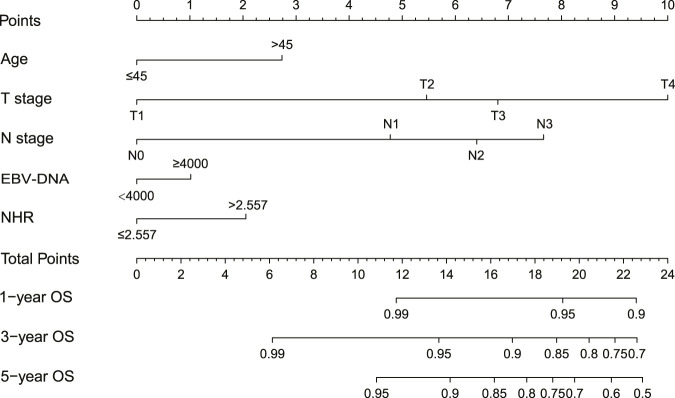
Nomogram of the integrated prognostic model for individualized 1-, 3-, and 5-year OS prediction. Abbreviations: NHR, neutrophil to high-density lipoprotein ratio; OS, overall survival.

Based on the independent prognostic factors identified by multivariate regression analysis, a nomogram integrating age, T stage, N stage, and NHR was constructed to predict 1-, 3-, and 5-year OS (Figure 2). Given that EBV-DNA is a well-established prognostic factor for NPC in line with clinical practice and prior researches, we integrated it as a key component into the nomogram. Individual patient scores are summed across factors to estimate survival probabilities. For instance, a 40-year-old patient with T2, N1, NHR >2.557, EBV-DNA <4000 copies/mL would have a total score of 12.286 points, correlating with estimated 1-year, 3-year, and 5-year OS probabilities of 99%, 96%, and 93%, respectively.

### Assessment of predictive performance of the prognostic model

The integrated nomogram showed discriminative ability with a C-index of 0.696 (95% CI: 0.654–0.738), numerically higher in absolute value than that of TNM staging 0.648 (95% CI: 0.583–0.713). Yet the DeLong test for comparing the nomogram with the TNM staging system yielded a P-value of 0.097, which indicated no statistically significant difference between these two prognostic approaches, while 1000-time bootstrapping validation yielded a corrected C-index of 0.706 (95% CI: 0.566–0.840) for the nomogram.

**FIGURE 3:**
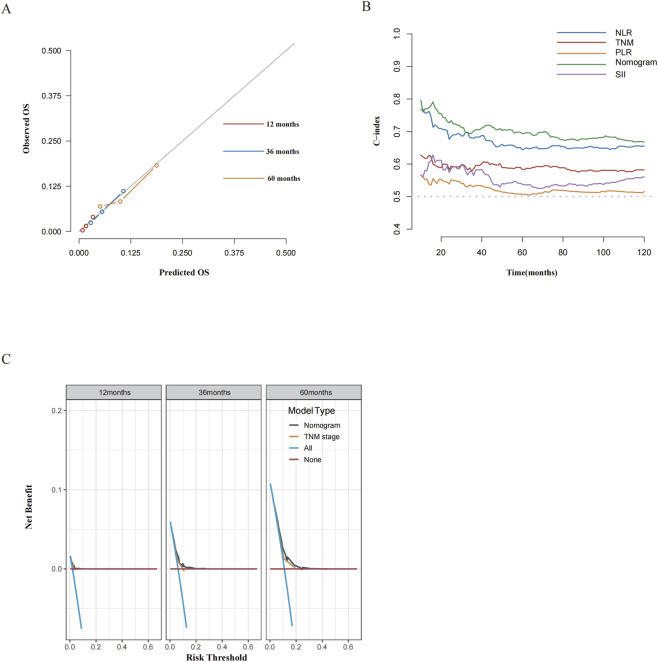
Assessment of predictive performance of the prognostic model. **(A)** Calibration plot of the nomogram model at 1-, 3-, and 5-year OS. **(B)** Time-dependent ROC curves compared the predictive accuracy of the integrated nomogram, the traditional TNM staging system, and other inflammatory biomarkers including NLR, PLR, and SII. **(C)** DCA curves compared the net benefit rate of the current model and the traditional TNM stage. Abbreviations: OS, overall survival; AUC, area under the curve; TNM, tumor-node-metastasis.

Calibration plots demonstrated strong agreement between predicted and observed OS at multiple time points (Figure 3A). Confirmed by tROC analysis was that the integrated nomogram exhibited slightly higher prognostic accuracy compared with the TNM staging system and other inflammatory biomarkers, including NLR, PLR, and SII (Figure 3B). DCA indicated greater net benefit of the nomogram versus TNM staging individually (Figure 3C).

## Discussion

Given the inherent limitations of the traditional TNM staging system in precise prognostic stratification for NPC patients treated with CCRT, to the best of our knowledge, this is the largest detailed study to explore the prognostic value of the tumor-related inflammatory metabolic marker, NHR, in a substantial cohort of such patients (Li et al., 2021). Derived from the neutrophil count and high-density lipoprotein level, the NHR encapsulates both systemic inflammatory status and metabolic status, thereby potentially offering additional insights into tumor outcomes. A higher pre-treatment level of NHR has been identified as being associated with poorer prognosis. The integration of NHR with clinical parameters (age, T stage, and N stage) into a comprehensive nomogram yielded enhanced prognostic precision for NPC patients. Compared to the standard TNM staging, this refined model yields moderately improved accuracy in survival predictions, thereby empowering clinicians to implement individualized management and more effective risk-based stratification within the CCRT setting.

NPC is biologically highly heterogeneous, with notably different outcomes observed among patients at the same stage receiving similar CCRT regimens (Au et al., 2018; Lee et al., 2019; Chen et al., 2019). While sophisticated molecular techniques like gene examination and liquid biopsy have emerged to interpret NPC’s molecular mechanisms and heterogeneity, their clinical application remains limited due to high costs and complex testing procedures (Liu et al., 2012; Lin et al., 2014). The traditional anatomical TNM staging system alone is insufficient for accurate prognosis prediction, highlighting the urgent need for additional efficient biomarkers that are affordable, easily accessible, and readily applicable in clinical practice (OuYang et al., 2015; Pan et al., 2016b).

The biological mechanisms underlying NHR’s prognostic value likely involve complex interactions between nutrition, inflammation, and tumor immunity. Neutrophils, as mediators of systemic inflammation, play crucial roles in tumor progression. Elevated neutrophil counts often reflect enhanced inflammatory responses, which can promote tumor growth and metastasis (Coffelt et al., 2016). Conversely, HDL exerts pivotal anti-inflammatory and antioxidant effects, regulating systemic inflammatory homeostasis and the tumor microenvironment (Kim et al., 2024; Zhou et al., 2018). Lipid metabolic dysregulation and inflammatory imbalance can compromise treatment outcomes through multiple mechanisms, including reduced treatment tolerance, decreased radiation sensitivity, and impaired immune function. The combination of these factors in NHR may provide a more comprehensive assessment of a patient’s condition than either component alone (Liu et al., 2019).

Our multivariate analysis identified several other independent prognostic factors, including age ≤45 years, T stage (T3 and T4), N stage (N1, N2, and N3). Age has been consistently recognized as an important prognostic factor in NPC, with younger patients showing better survival outcomes (Xiao et al., 2013). This age-related prognostic difference may be attributed to better treatment tolerance, stronger immune function, and fewer comorbidities in younger patients. Advanced T and N stages were strongly associated with poorer survival, reflecting the impact of tumor extent and regional spread on prognosis (Mao et al., 2009). These findings align with previous studies and underscore the continued relevance of anatomical staging in risk assessment.

Nevertheless, several limitations should be considered when interpreting our results. Firstly, as a retrospective single-center study lacking multicenter validation, selection bias cannot be excluded, which also restricts the generalizability of our findings and the clinical utility of the constructed nomogram. Secondly, potential clinical confounders including smoking status and performance status were not included in our analysis, which may have affected the robustness of our prognostic findings. Thirdly, we only assessed pre-treatment NHR values, while unexplored dynamic changes during treatment might provide additional prognostic information. Fourthly, the absence of Disease-free survival (DFS) analyses limits a comprehensive assessment of disease recurrence patterns in NPC patients. Fifthly, external validation in independent cohorts is required to confirm our findings, a pivotal step that verifies the robustness and generalizability of NHR’s prognostic value and the constructed nomogram, and further facilitates the translational application of our research results to clinical practice for the broader NPC patient population. Furthermore, the potential influence of various clinical conditions on NHR values requires further investigation, including the unaddressed impact of comorbidities such as diabetes on HDL levels. Future prospective multicenter studies are warranted to validate the prognostic value of NHR in NPC, explore the impact of dynamic changes in NHR levels on patient prognosis, address the inherent limitations of the present study, and further elucidate the underlying biological mechanisms mediating the prognostic significance of NHR in this disease.

## Conclusion

This study validates NHR as an innovative, readily accessible, cost-efficient, and minimally invasive indicator for predicting prognosis in NPC patients undergoing CCRT. Furthermore, prognostic models based on NHR have demonstrated slightly higher predictive performance in comparison to conventional staging approaches. By utilizing the NHR-based scoring system, oncologists can enhance the precision of survival outcome forecasts, thereby facilitating the development of optimal management strategies for NPC patients both prior to and following CCRT.

## Data Availability

All relevant data supporting the findings of this study have been deposited in the Research Data Deposit (RDD) repository The permanent access link to the repository is: http://183.236.15.75:19800/UserHome/ProjectAuthorConfirm.aspx?ProjectID=6379&ProjectAuthorID=C5A02B36E29037D7.

## References

[B1] Au K. H. Ngan R. K. C. Ng A. W. Y. Poon D. M. C. Ng W. T. Yuen K. T. (2018). Treatment outcomes of nasopharyngeal carcinoma in modern era after intensity modulated radiotherapy (IMRT) in Hong Kong: a report of 3328 patients (HKNPCSG 1301 study). Oral Oncol. 77, 16–21. 10.1016/j.oraloncology.2017.12.004 29362121

[B2] Blanchard P. De Felice F. Chua M. L. K. (2025). Advances in individualization of systemic treatment for locoregionally advanced nasopharyngeal carcinoma: a systematic review. ESMO Open 10 (4), 104513. 10.1016/j.esmoop.2025.104513 40138744 PMC11985003

[B3] Chen L. Zhang Y. Lai S. Z. Li W. F. Hu W. H. Sun R. (2019). 10-Year results of therapeutic ratio by intensity-modulated radiotherapy *versus* two-dimensional radiotherapy in patients with nasopharyngeal carcinoma. Oncologist 24 (1), e38–e45. 10.1634/theoncologist.2017-0577 30082487 PMC6324627

[B4] Chiang C. L. Guo Q. Ng W. T. Lin S. Ma T. S. W. Xu Z. (2021). Prognostic factors for overall survival in nasopharyngeal cancer and implication for TNM staging by UICC: a systematic review of the literature. Front. Oncol. 11, 703995. 10.3389/fonc.2021.703995 34540670 PMC8445029

[B5] Coffelt S. B. Wellenstein M. D. de Visser K. E. (2016). Neutrophils in cancer: neutral no more. Nat. Rev. Cancer 16 (7), 431–446. 10.1038/nrc.2016.52 27282249

[B6] Grivennikov S. I. Greten F. R. Karin M. (2010). Immunity, inflammation, and cancer. Cell. 140 (6), 883–899. 10.1016/j.cell.2010.01.025 20303878 PMC2866629

[B7] Hanahan D. Weinberg R. A. (2011). Hallmarks of cancer: the next generation. Cell 144 (5), 646–674. 10.1016/j.cell.2011.02.013 21376230

[B8] Huang Z. Fu Z. Huang W. Huang K. (2020). Prognostic value of neutrophil-to-lymphocyte ratio in sepsis: a meta-analysis. Am. J. Emerg. Med. 38 (3), 641–647. 10.1016/j.ajem.2019.10.023 31785981

[B9] Juarez-Vignon Whaley J. J. Afkhami M. Sampath S. Amini A. Bell D. Villaflor V. M. (2023). Early stage and locally advanced nasopharyngeal carcinoma treatment from present to future: where are we and where are we going? Curr. Treat. Options Oncol. 24 (7), 845–866. 10.1007/s11864-023-01083-2 37145382 PMC10271909

[B10] Kim S. Kim G. Cho S. H. Oh R. Kim J. Y. Lee Y. B. (2024). Association between total cholesterol levels and all-cause mortality among newly diagnosed patients with cancer. Sci. Rep. 14 (1), 58. 10.1038/s41598-023-50931-6 38168969 PMC10761709

[B11] Lee V. H. Kwong D. L. Leung T. W. Choi C. W. O'Sullivan B. Lam K. O. (2019). The addition of pretreatment plasma epstein-barr virus DNA into the eighth edition of nasopharyngeal cancer TNM stage classification. Int. J. Cancer 144 (7), 1713–1722. 10.1002/ijc.31856 30192385

[B12] Li J. Wu Y. L. Li W. F. Ma J. (2021). Neutrophil to apolipoprotein A-I ratio as an independent indicator of locally advanced nasopharyngeal carcinoma. Laryngoscope Investig. Otolaryngol. 6 (5), 1049–1061. 10.1002/lio2.660 34667849 PMC8513451

[B13] Lin D. C. Meng X. Hazawa M. Nagata Y. Varela A. M. Xu L. (2014). The genomic landscape of nasopharyngeal carcinoma. Nat. Genet. 46 (8), 866–871. 10.1038/ng.3006 24952746

[B14] Liu N. Chen N. Y. Cui R. X. Li W. F. Li Y. Wei R. R. (2012). Prognostic value of a microRNA signature in nasopharyngeal carcinoma: a microRNA expression analysis. Lancet Oncol. 13 (6), 633–641. 10.1016/S1470-2045(12)70102-X 22560814

[B15] Liu M. Z. Fang S. G. Huang W. Wang H. Y. Tian Y. M. Huang R. D. (2019). Clinical characteristics and prognostic value of pre-retreatment plasma epstein-barr virus DNA in locoregional recurrent nasopharyngeal carcinoma. Cancer Med. 8 (10), 4633–4643. 10.1002/cam4.2339 31268626 PMC6712460

[B16] Liu Q. Ma L. Ma H. Yang L. Xu Z. (2022). Establishment of a prognostic nomogram for patients with locoregionally advanced nasopharyngeal carcinoma incorporating clinical characteristics and dynamic changes in hematological and inflammatory markers. Front. Oncol. 12, 1032213. 10.3389/fonc.2022.1032213 36387244 PMC9643849

[B17] Liu Y. Liu X. Sun S. Han Y. Feng M. Zhang Y. (2024). Evidence of being cured for nasopharyngeal carcinoma: results of a multicenter patient-based study in China. Lancet Reg. Health West Pac 49, 101147. 10.1016/j.lanwpc.2024.101147 39149139 PMC11325080

[B18] Liu Q. Wang H. Chen Z. Xiong J. Huang Y. Zhang S. (2025). Global, regional, and national epidemiology of nasopharyngeal carcinoma in middle-aged and elderly patients from 1990 to 2021. Ageing Res. Rev. 104, 102613. 10.1016/j.arr.2024.102613 39626854

[B19] Mai H. Q. Chen Q. Y. Chen D. Hu C. Yang K. Wen J. (2023). Toripalimab plus chemotherapy for recurrent or metastatic nasopharyngeal carcinoma: the JUPITER-02 randomized clinical trial. JAMA 330 (20), 1961–1970. 10.1001/jama.2023.20181 38015220 PMC10685882

[B20] Mantovani A. Allavena P. Sica A. Balkwill F. (2008). Cancer-related inflammation. Nature 454 (7203), 436–444. 10.1038/nature07205 18650914

[B21] Mao Y. P. Xie F. Y. Liu L. Z. Sun Y. Li L. Tang L. L. (2009). Re-evaluation of 6th edition of AJCC staging system for nasopharyngeal carcinoma and proposed improvement based on magnetic resonance imaging. Int. J. Radiat. Oncol. Biol. Phys. 73 (5), 1326–1334. 10.1016/j.ijrobp.2008.07.062 19153016

[B22] Miao J. Wang L. Tan S. H. Li J. G. Yi J. Ong E. H. W. (2022). Adjuvant capecitabine following concurrent chemoradiotherapy in locoregionally advanced nasopharyngeal carcinoma: a randomized clinical trial. JAMA Oncol. 8 (12), 1776–1785. 10.1001/jamaoncol.2022.4656 36227615 PMC9562101

[B23] OuYang P. Y. Zhang L. N. Lan X. W. Xie C. Zhang W. W. Wang Q. X. (2015). The significant survival advantage of female sex in nasopharyngeal carcinoma: a propensity-matched analysis. Br. J. Cancer 112 (9), 1554–1561. 10.1038/bjc.2015.70 25742485 PMC4453682

[B24] Pan J. J. Ng W. T. Zong J. F. Lee S. W. M. Choi H. C. W. Chan L. L. K. (2016a). Prognostic nomogram for refining the prognostication of the proposed 8th edition of the AJCC/UICC staging system for nasopharyngeal cancer in the era of intensity-modulated radiotherapy. Cancer. 122 (21), 3307–3315. 10.1002/cncr.30198 27434142 PMC5524130

[B25] Pan J. J. Ng W. T. Zong J. F. Chan L. L. K. O'Sullivan B. Lin S. J. (2016b). Proposal for the 8th edition of the AJCC/UICC staging system for nasopharyngeal cancer in the era of intensity-modulated radiotherapy. Cancer. 122 (4), 546–558. 10.1002/cncr.29795 26588425 PMC4968037

[B26] Peng Q. Zhan C. Shen Y. Xu Y. Ren B. Feng Z. (2024). Blood lipid metabolic biomarkers are emerging as significant prognostic indicators for survival in cancer patients. BMC Cancer 24 (1), 1549. 10.1186/s12885-024-13265-8 39695484 PMC11657272

[B27] Rai P. Lakhani D. A. Agarwal A. Bhatt A. A. (2025). The 9th version of the AJCC staging system for nasopharyngeal carcinoma: a guide for radiologists. AJR Am. J. Roentgenol. 225 (2), e2533016. 10.2214/AJR.25.33016 40304669

[B28] Shi K. Hou J. Zhang Q. Bi Y. Zeng X. Wang X. (2023). Neutrophil-to-high-density-lipoprotein-cholesterol ratio and mortality among patients with hepatocellular carcinoma. Front. Nutr. 10, 1127913. 10.3389/fnut.2023.1127913 37215223 PMC10198653

[B29] Tang L. L. Guo R. Zhang N. Deng B. Chen L. Cheng Z. B. (2022). Effect of radiotherapy alone vs radiotherapy with concurrent chemoradiotherapy on survival without disease relapse in patients with low-risk nasopharyngeal carcinoma: a randomized clinical trial. JAMA 328 (8), 728–736. 10.1001/jama.2022.13997 35997729 PMC9399866

[B30] Templeton A. J. McNamara M. G. Seruga B. Vera-Badillo F. E. Aneja P. Ocaña A. (2014). Prognostic role of neutrophil-to-lymphocyte ratio in solid tumors: a systematic review and meta-analysis. J. Natl. Cancer Inst. 106 (6), dju124. 10.1093/jnci/dju124 24875653

[B31] Wang J. Zhou D. Dai Z. Li X. (2021). Association between systemic immune-inflammation index and diabetic depression. Clin. Interv. Aging 16, 97–105. 10.2147/CIA.S285000 33469277 PMC7810592

[B32] Wang R. Dai W. Gong J. Huang M. Hu T. Li H. (2022). Development of a novel combined nomogram model integrating deep learning-pathomics, radiomics and immunoscore to predict postoperative outcome of colorectal cancer lung metastasis patients. J. Hematol. Oncol. 15 (1), 11. 10.1186/s13045-022-01225-3 35073937 PMC8785554

[B33] Xiao G. Cao Y. Qiu X. Wang W. Wang Y. (2013). Influence of gender and age on the survival of patients with nasopharyngeal carcinoma. BMC Cancer 13, 226. 10.1186/1471-2407-13-226 23642234 PMC3672024

[B34] Yuan X. Feng H. Huang H. Li J. Wu S. Yuan Y. (2023). Systemic immune-inflammation index during treatment predicts prognosis and guides clinical treatment in patients with nasopharyngeal carcinoma. J. Cancer Res. Clin. Oncol. 149 (1), 191–202. 10.1007/s00432-022-04506-z 36595043 PMC9889477

[B35] Zhang Y. Chen L. Hu G. Q. Zhang N. Zhu X. D. Yang K. Y. (2019). Gemcitabine and cisplatin induction chemotherapy in nasopharyngeal carcinoma. N. Engl. J. Med. 381 (12), 1124–1135. 10.1056/NEJMoa1905287 31150573

[B36] Zhou P. Li B. Liu B. Chen T. Xiao J. (2018). Prognostic role of serum total cholesterol and high-density lipoprotein cholesterol in cancer survivors: a systematic review and meta-analysis. Clin. Chim. Acta. 477, 94–104. 10.1016/j.cca.2017.11.039 29223765

